# Two novel genomic regions associated with fearfulness in dogs overlap human neuropsychiatric loci

**DOI:** 10.1038/s41398-018-0361-x

**Published:** 2019-01-17

**Authors:** R. Sarviaho, O. Hakosalo, K. Tiira, S. Sulkama, E. Salmela, M. K. Hytönen, M. J. Sillanpää, H. Lohi

**Affiliations:** 1 Department of Veterinary Biosciences, University of Helsinki, 00014 Helsinki, Finland; 2 Research Programs Unit, Molecular Neurology, University of Helsinki, 00014 Helsinki, Finland; 3 The Folkhälsan Institute of Genetics, 00290 Helsinki, Finland; 4 Equine and Small Animal Medicine, University of Helsinki, Helsinki, Finland; 5 Department of Biosciences, University of Helsinki, Helsinki, Finland; 6 Department of Mathematical Sciences and Biocenter Oulu, University of Oulu, Oulu, Finland

## Abstract

Anxiety disorders are among the leading health issues in human medicine. The complex phenotypic and allelic nature of these traits as well as the challenge of establishing reliable measures of the heritable component of behaviour from the associated environmental factors hampers progress in their molecular aetiology. Dogs exhibit large natural variation in fearful and anxious behaviour and could facilitate progress in the molecular aetiology due to their unique genetic architecture. We have performed a genome-wide association study with a canine high-density SNP array in a cohort of 330 German Shepherds for two phenotypes, fear of loud noises (noise sensitivity) and fear of strangers or in novel situations. Genome-widely significant loci were discovered for the traits on chromosomes 20 and 7, respectively. The regions overlap human neuropsychiatric loci, including 18p11.2, with physiologically relevant candidate genes that contribute to glutamatergic and dopaminergic neurotransmission in the brain. In addition, the noise-sensitivity locus includes hearing-related candidate genes. These results indicate a genetic contribution for canine fear and suggest a shared molecular aetiology of anxiety across species. Further characterisation of the identified loci will pave the way to molecular understanding of the conditions as a prerequisite for improved therapy.

## Introduction

Anxiety disorders include a large spectrum of heterogeneous conditions and rank among the most common health concerns in human medicine with a lifetime prevalence of > 15%^1^. Better understanding of the biological background of anxiety is essential due to current lack of efficient treatment and therapy. Anxiety disorders are heritable but genetically complex, which emphasizes the need of various approaches for identifying genetic determinants, including comparative physiologically relevant animal models^1,2^.

Since the annotation of their genome, domestic dogs have emerged as powerful models for genetic studies due to their unique genetic architecture combined with a large body size, extensive breed variety and the presence of hundreds of naturally occurring morphological, behavioural and disease phenotypes^3^. Over 230 genes have been recently implicated in various canine Mendelian disorders and traits^4,5^ and successful examples include also discoveries for several complex phenotypes such as cancer^6^, autoimmune disease^7^ and size^8^. Although discoveries in canine behaviour are still rare and largely suggestive, candidate loci and genes have been reported for obsessive-compulsive disorder^9,10^, social behaviour^11,12^, fear and aggression^13^. The relative scarcity of the genetic discoveries in behaviour is likely due to the complex phenotypic and allelic nature of the traits, as well as the challenge of establishing reliable measures of the heritable component of behaviour from the associated environmental factors^14^.

Fear is an evolutionarily important and conserved emotional state crucial for fitness and survival in all animals. Increased fearfulness in dogs can, however, cause several behavioural problems. Many anxiety-related behavioural conditions, such as generalized anxiety disorders, phobias and separation anxiety are seen and diagnosed in dogs and comprise substantial welfare issues^15^. Fear often motivates aggressiveness^16^, and bite injuries resulting from dog or human-directed aggression can be considered a serious health concern. In dogs, fearfulness can be categorised based on the object and the situation in which the behaviour occurs into social and non-social fearfulness^17^. The social category includes fear of unfamiliar people and dogs, whereas the non-social category includes fear of different objects such as new situations, loud noises (known as noise phobia or noise sensitivity (NS)), heights, or shiny and slippery floors^16^.

Fear of loud noises, such as thunder, gunshot or fireworks, is often referred to as noise phobia because of the strong panic reactions that are seen in some dogs^18^. However, in many cases the actual behavioural reactions are milder, and fear of loud noises is thus considered (and referred to) as NS^19^. Although NS and general fearfulness are somewhat overlapping, there are many otherwise non-fearful dogs with extreme NS^20^, and NS is considered an independent veterinary behavioural diagnosis^21,22^. Despite the high prevalence of NS across breeds that varies from 25 to 49%^15,16,23^, the pathophysiology is not known. In humans, sound sensitivity includes various conditions such as hyperacusis, phonophobia and misophonia^24,25^, which are sensitivities to certain frequencies, intensities or types of sounds, associated with strong feelings of dislike, fear or even aggression. Although environmental factors such as trauma may be responsible for some noise-sensitivity cases, both human and canine noise sensitivities have high heritability estimates (0.40 and 0.56, respectively), suggesting a substantial genetic component to this trait^26,27^.

Fear of strangers and of new situations are highly correlated in dogs^16^. Both traits are considered to be signs of generalized fear^21,22^. Dogs vary in their response to novel situations and unfamiliar people, with reactions ranging from extreme fearfulness to high sociability and curiosity^28^. Environmental factors such as lack of socialization, poor maternal care and aversive learning are known risk factors for canine fear^16,28,29^. On the other hand, high heritability estimates have been reported for fearfulness (from 0.36 to 0.49), suggesting a substantial genetic component to this trait^30,31^.

As a part of our larger effort to understand the molecular aetiology of canine anxiety^28,32,33^, we have performed here a genome-wide association study of 330 German Shepherds and identified novel loci for NS and fearfulness on chromosomes 20 and 7, respectively. These two loci overlap regions that have been mapped for human neuropsychiatric traits and harbour functional candidate genes that affect glutamatergic and dopaminergic neurotransmission and the hearing system.

## Materials and methods

### Study cohorts

Privately owned Finnish German Shepherd dogs originating from both working and show breeding lines were recruited to the study using a previously-published and validated owner-completed behavioural survey focusing on fearfulness and NS^16,20^. The breed was chosen for its known large variation in reacting to loud noises, strangers and novel situations (shyness–boldness personality). Besides detailed demographics, the owners were asked about their dogs’ reactions in various specific situations such as when meeting unfamiliar people, in new situations, or when hearing loud noises (thunder, fireworks and gunshot). The owners were also asked to specify the observed behaviour and its frequency.

**Fig. 1:**
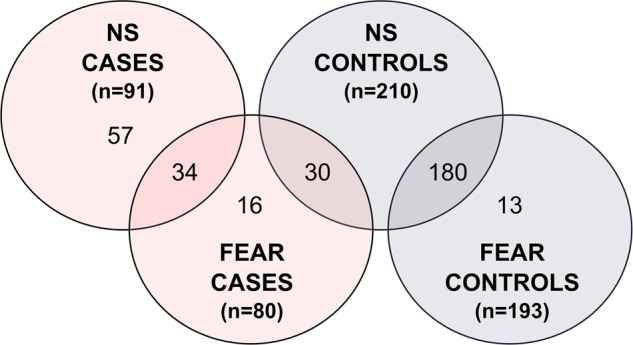
Distribution of the 330 dogs in the genetic analysis of fear and NS. The 330 German Shepherd dogs included in the study were categorised into noise sensitivity, fear and the respective control groups. Several dogs were included in two different groups

Altogether 330 German Shepherd dogs were phenotyped (Fig. 1) using our behavioural questionnaire. For the NS cohort, dogs were categorized to cases and controls based on the noise reactivity score (NRS)^16^. The NRS describes the frequency and intensity of a fearful reaction towards loud noise and was calculated as follows: (sum of fearful behavioural reactions to fireworks) × (frequency of fear reaction to fireworks) + (sum of fearful behavioural reactions to thunder) × (frequency of fear reaction to thunder) + (sum of fearful behavioural reactions to gunshot) × (frequency of fear reaction to gunshot) (Supplementary Table 1). The range of possible NRS values is from 0 to 156 with intervals of 1. Dogs showing the most severe, generalized and frequent fear reactions gain the highest scores. The NRS of the control dogs in the study cohort was 0 and the dogs were over 5 years old. The owners of the mildest cases (scores 1–3) were contacted by phone or email to confirm the NS status. The age of onset was available only for 32 (out of 91) NS cases. In these dogs the age of onset varied from three months to ten years with a median age of onset of 4 years. In a larger study cohort (*N* > 3000 dogs) only 15.1% of the dogs were reported with NS onset of over 5 years^16^. According to our behavioural survey data, the owners of eight controls were not aware how their dogs react to gunshot (these dogs were not afraid of thunder or fireworks). This is only 3.8% of all our controls. Additionally, there were altogether 13 cases without reported exposure to gunshots.

For the fear cohort, the dogs were categorized according to a fear reaction score (FRS), which was based on the dogs’ reactions (intensity and frequency) towards unfamiliar people and new situations. Fear of strangers and new situations are highly correlated. Our previous study shows that the more often the dog shows fearful behaviour towards strangers, the more often it also shows fear in new situations^16^. Fear towards strange dogs was not included in the score for fearfulness, since it is heavily affected by dogs’ experiences of other dogs and their level of training. The FRS was calculated as an average score as follows: ((sum of fearful behavioural reactions when meeting unfamiliar people, where withdrawal was multiplied by 5) × (frequency of fear reactions when meeting unfamiliar people) + (sum of fearful behavioural reactions in novel situations) × (frequency of fear reactions in novel situations)) × 0.5 (Supplementary Table 2). Avoidance was emphasized in the scoring by a factor of 5 because it is a common, clear and strong fear reaction, easily recognized by owners^34^. Avoidance is the only behaviour among the possible fear reactions where the dog can be interpreted to flee the object of fear, and where the dog’s sole motivation for the observed behaviour can be interpreted to be fear for that object. For behaviours such as barking or growling, the motivation can also be protective. Additionally, avoidance or withdrawal is the most frequent option marked in our questionnaire according to our previous study^16^. The range of possible FRS values is from 0 to 42 with intervals of 0.5. The FRS value for control dogs in the fear study cohort was 0 and none showed fear towards loud noise. All case and control dogs were required to be older than one year, as the canine personality has been found to be stable from one year onwards^35^.

For the Extended Bayesian Lasso analysis, the NRS and FRS scores were additionally categorized into five groups. In this categorization all control dogs had a value of 0, while case dogs were divided into four categories with even intervals.

### Samples

EDTA-blood samples were collected from 330 privately owned German Shepherd dogs for DNA and the samples were stored at −20 °C until genomic DNA was extracted using a semi-automated Chemagen extraction robot (PerkinElmer Chemagen Technologie). DNA concentration was determined either with a Qubit 3.0 Fluorometer (Thermo Fisher Scientific) or a NanoDrop ND-1000 UV/Vis Spectrophotometer. Sample collection was ethically approved by the Animal Ethics Committee of State Provincial Office of Southern Finland, Hämeenlinna, Finland (ESAVI/6054/04.10.03/ 2012).

### Genome-wide association study

Altogether 330 German Shepherds were genotyped using Illumina’s Canine HD (173k) SNP arrays (Fig. 1). A total of 91 cases and 210 controls were included in the analysis of NS and 80 cases and 193 controls in fear (Fig. 1). The same set of control dogs was partially utilized in the fear study, excluding the dogs that had any fear reactions. In addition, thirteen control dogs that were too young to be included as controls in the NS study were used as controls in the fear study (Fig. 1). The genotype data were filtered with a SNP call rate of > 90%, array call rate of > 90%, minor allele frequency of > 1% and by using a Hardy–Weinberg equilibrium of *P* ≥ 1 × 10^–8^. No individual dogs were removed and 94 552 (NS) or 94 434 (fear) SNPs remained for analysis. Genotyping data was analysed using various statistical methods including single-locus association analysis (PLINK)^36^, single-locus mixed model approaches of Grammar-gamma and FASTA implemented in R package GenABEL^37^ and a multilocus Extended Bayesian Lasso (EBL) method^38–40^. The PLINK and GenABEL analyses were performed using a quantitative trait approach for the given phenotype measurements. In EBL, an ordinal categorical phenotype of five classes was used.

**Fig. 2:**
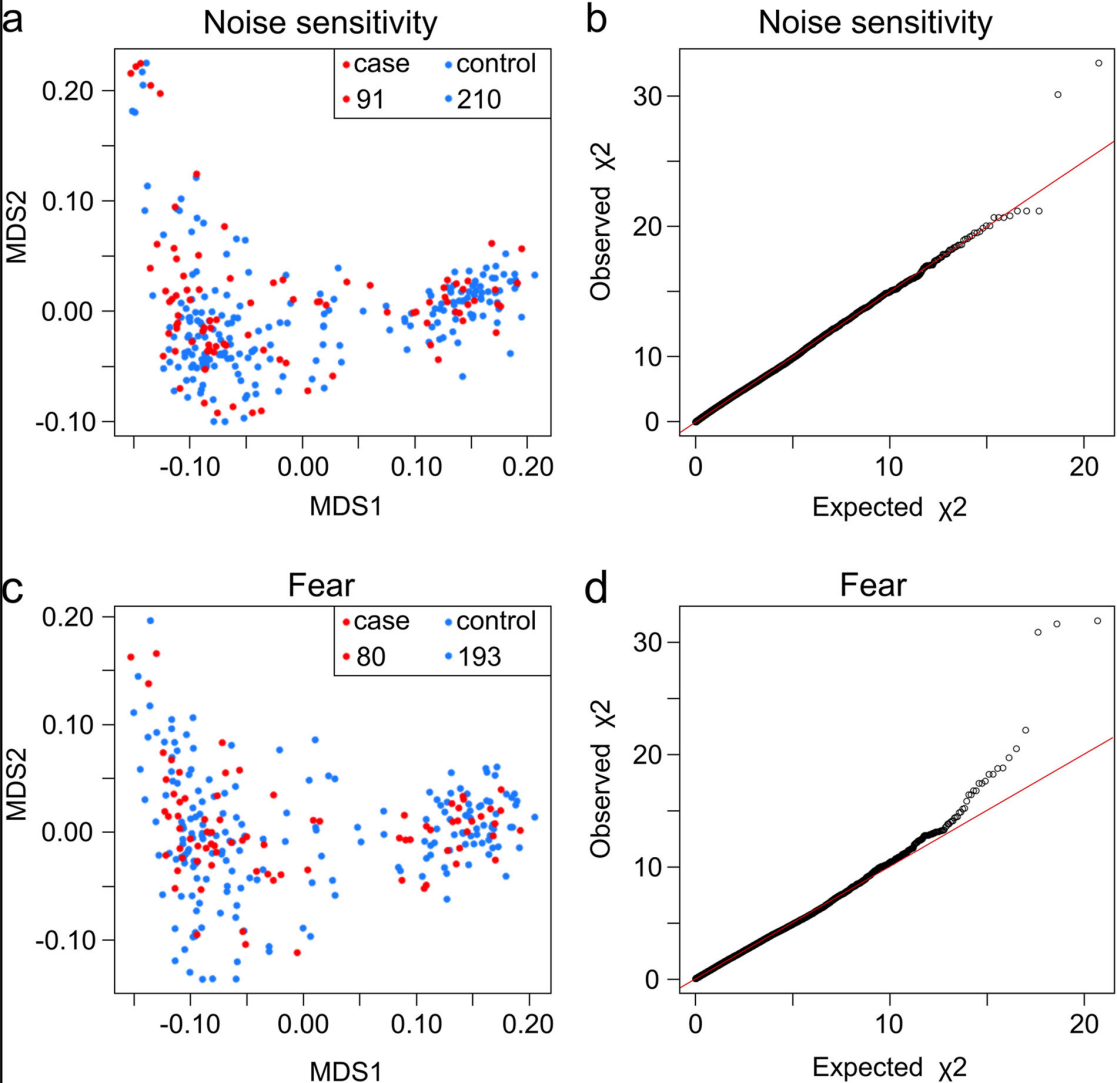
Multidimensional scaling and quantile–quantile plots for the NS and fear cohorts. **a** MDS plot for NS **b** q–q plot for NS (*λ* = 1.022). **c** MDS plot for fear. **d** q–q plot for fear (*λ* = 1.020) The study cohorts include show and working line German Shepherds, which are seen in separate genetic clusters. Working line dogs are grouped on the left side of the plot while show line dogs are seen on the right, with dogs with a mixed heritage in the middle

Existence of potential confounding signals due to cryptic relatedness and population stratification was evaluated from multidimensional scaling (MDS) and quantile–quantile (q–q) plots in the study populations (Fig. 2). The data were adjusted for relatedness and population stratification either by including a polygenic effect term with marker-estimated relationship matrix to the statistical model (GenABEL)^41^ or adjusting by genomic control approach (PLINK)^42^. In PLINK and GenABEL, the statistical significance of loci was tested using Bonferroni-corrected threshold value accounting for multiple testing. The significance level after correcting for multiple testing was 5.4E-07 in the genome-wide association analysis (GWAS) of NS (94,552 tests) and 5.4E-07 in the GWAS of fear (94,434 tests).

In the multilocus EBL model, the false signals due to cryptic relatedness do not need to be separately corrected^39,43–45^. Following Kärkkäinen and Sillanpää 2012^39^, to reduce number of explanatory variables before EBL analysis, we performed sure-independence-screening^46^, where 5000 best-ranking SNPs were selected out of the original SNPs. Estimation in EBL was performed using in-house C + + /R program implementing Generalized Expectation-Maximization algorithm to find Bayesian point-estimates for quantitative trait locus (QTL) effects^39,40,47^. In the Bayesian approach, priors need to be specified. Hyper-parameters of the prior distributions were tuned for this specific data set by using the values that seemed to provide reasonable-looking outputs while not being exceedingly sensitive to small changes of given parameter values.

Throughout the study, the CanFam 3.1 annotation was used.

## Results

**Fig. 3:**
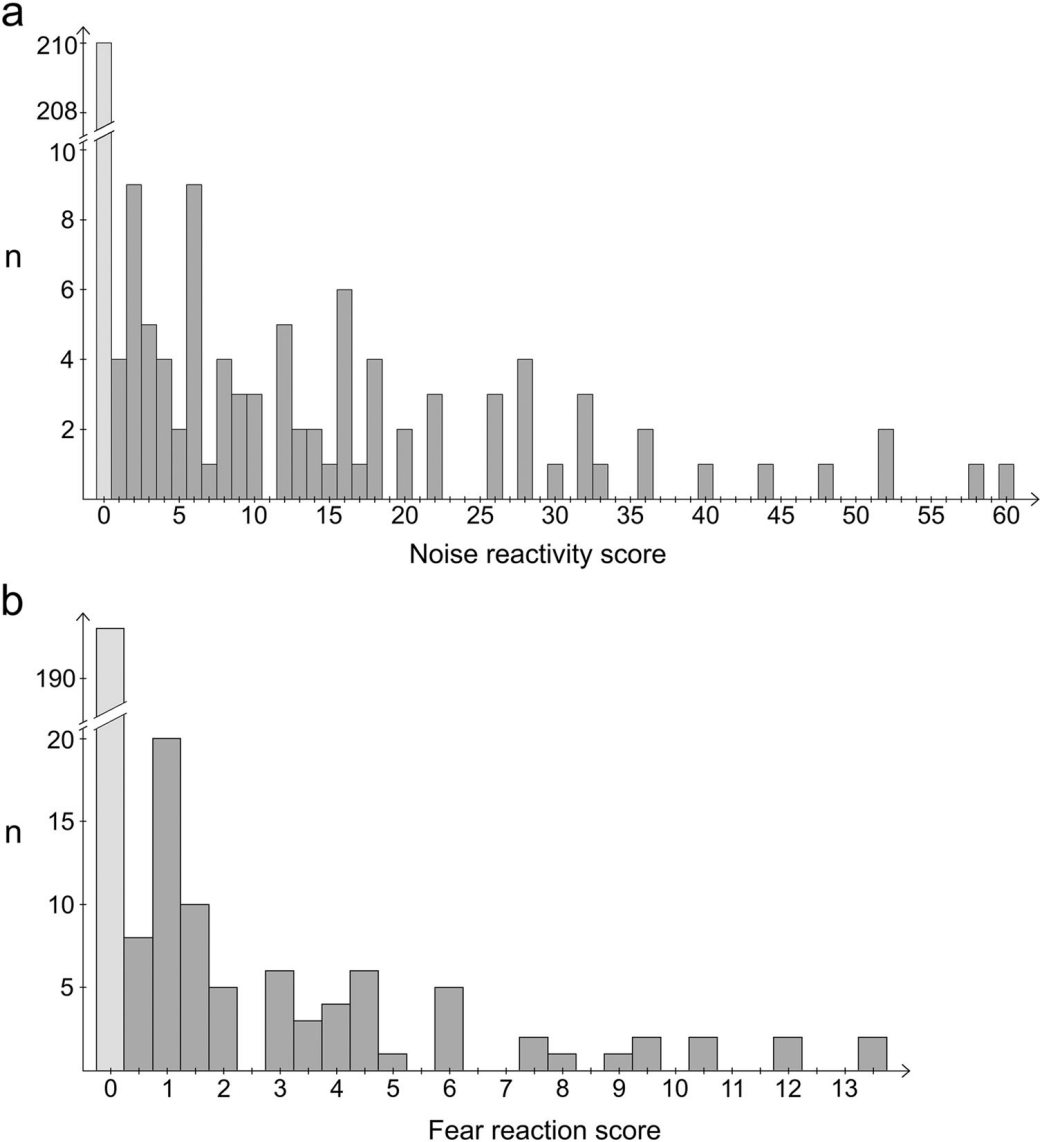
Distribution of the noise reactivity and fear reaction scores. The noise (**a**) and fear (**b**) reactivity scores in the noise sensitivity (*n* = 301) and fear (*n* = 273) cohorts were distributed from 0 to 60 or from 0 to 13.5, respectively. In both cohorts, the dogs with a score of 0 were used as controls

Altogether 330 German Shepherds were carefully phenotyped for two behavioural traits, NS and fear of strangers or novel situations (fear), using quantitative categorical “noise reactivity” and “fear reaction” scores (NRS and FRS). The scores were established in order to be able to evaluate the intensity of the phenotype. The NRS values of NS cases varied between 1 and 60 with a median value of 12 and an average of 15.5 while controls had a value of 0 (Fig. 3a). In dogs that were used as cases in the fear cohort, FRS varied between 0.5 and 13.5 with a median value of 2 and an average of 3.5, while all controls had a value of 0 (Fig. 3b). The study cohorts included both show and working line German Shepherds, which clustered separately in MDS plots (Fig. 2a, c). The genomic inflation factors (*λ*) were 1.022 and 1.020 for the NS and fear cohorts, respectively, suggesting low levels of population stratification (Fig. 2b, d).

The phenotypic scores were not normally distributed (Fig. 3) and therefore, the data were analysed using three different approaches. First, a quantitative analysis with the NRS and FRS scores was performed using PLINK and GenABEL, erroneously assuming a normal distribution. To further assess which findings replicate, a multilocus Extended Bayesian Lasso (EBL) analysis, not expecting a normal distribution, was performed for an ordinal categorical phenotype in five subgroups (0–4). Replication in categorical data analysis gives information on whether the earlier PLINK & GenABEL findings are false positive due to non-normal phenotype distribution, or true positive resulting from real phenotype associations.

### NS maps to chromosome 20

**Fig. 4:**
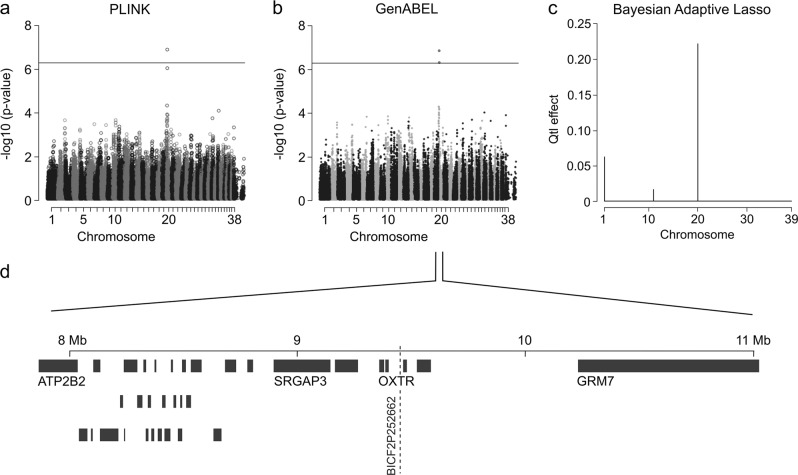
GWAS for NS. **a** A single-locus-analysed Manhattan plot (PLINK) indicates best *p*-values at chromosome 20 in a genome-wide analysis. **b** A Manhattan plot of a mixed model association analysis (GenABEL) indicates best *p*-values at chromosome 20 in a genome-wide analysis. **c** An Extended Bayesian Lasso method indicates the best signal (posterior QTL-effect) in chromosome 20. **d** The associated genomic region includes several known anxiety and hearing-related candidate genes

To map loci for NS, dogs were grouped to cases and controls based on the NRS (Fig. 3a). Altogether 301 German Shepherds were genotyped, including 91 cases and 210 control dogs. The quantitative PLINK and GenABEL GWA analyses revealed a statistically significant association on chromosome 20 after Bonferroni correction (Fig. 4a, b). The two best-associated SNPs with genome-wide significance span a region from 9,451,007 to 9,731,317 bp on CFA20 (Supplementary Table 3). The EBL analysis revealed the best signal in the same region (Fig. 4c), further supporting the fact that the association in chromosome 20 is true and not an artefact due to non-normal phenotype distribution. The locus includes several candidate genes for neuropsychiatric and hearing-related phenotypes, including the oxytocin receptor gene *OXTR*, a Rho GTPase gene *SRGAP3*, a metabotropic glutamate receptor gene *GRM7* and a plasma membrane calcium ATPase gene *ATP2B2*.

### Fear towards strangers and new situations maps to chromosome 7

**Fig. 5:**
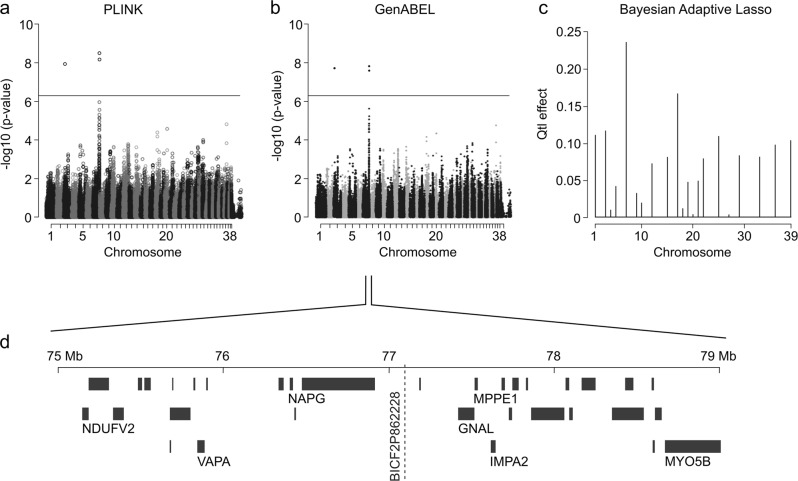
GWAS for fear. **a** A single-locus-analysed Manhattan plot (PLINK) indicates best *p*-values at chromosome 7 in a genome-wide analysis. **b** A Manhattan plot of a mixed model association analysis (GenABEL) indicates best *p*-values at chromosome 7 in a genome-wide analysis. **c** An Extended Bayesian Lasso method indicates the best signal (posterior QTL-effect) in chromosome 7. **d** The associated genomic region is syntenic to the human 18p11.2 locus that has been associated with various psychiatric disorders

To map loci for fear towards strangers and new situations, dogs were categorised to cases and controls based on the FRS (Fig. 3b). Examples of a fearful and a non-fearful dog in a behavioural test are shown in Supplementary Video 1. Altogether 273 German Shepherds were genotyped, including 80 cases and 193 control dogs. Both PLINK and GenABEL GWA analyses revealed a significant association on chromosome 7 after Bonferroni correction (Fig. 5a, b). The two best-associated SNPs span from 75,935,406 to 77,639,464 bp on CFA7 (Supplementary Table 4). The EBL analysis revealed several signals in various chromosomes, however, the best signal was seen in chromosome 7 (Fig. 5c), suggesting that the association found by frequentist approaches is true. The associated region is largely syntenic to a human 18p11.2 locus that has been linked to neuropsychiatric disorders. In addition, an isolated single SNP showing association was seen on chromosome 3 (BICF2G630701448, *p* = 1.93E-08).

The patterns of the background noise in the PLINK and GenABEL analyses are rather similar in both NS and fear analyses (Figs. 4, 5). We expect that this phenomenon is a consequence of the non-normal phenotype distribution, which shows up as similar background noise patterns. Since the Bayesian analyses support the PLINK and GenABEL signals in both traits, we believe our findings do not occur because of non-normality of phenotype distributions.

## Discussion

We have identified two novel loci for fearfulness in German Shepherd dogs. The significance of the findings is highlighted by the fact that these loci overlap genomic regions that include candidate genes that participate in the regulation of the neuronal glutamate and dopamine systems and have been linked to both neuropsychiatric illnesses and hearing defects. Although larger replication studies are necessary to confirm the findings, this study demonstrates the genetic susceptibility to fear in dogs and facilitates the search for underlying pathophysiology, which may be shared across species.

NS is a common trait in dogs with up to 50% of individuals across breeds suffering from it^16^. Noise-sensitive dogs react to sounds in several ways, most commonly by panting, pacing, by having their tails between their legs, trembling or escaping, but reactions may also include salivation, urination or destroying^16^. Dogs with NS have previously been suggested to be natural models for human panic disorder, exhibiting some physiological similarities and shared symptoms with human patients^18^. However, while panic disorder does not have a specific focus of fear, NS does, which composes a marked difference between the two disorders. A recent study suggested a slight change in the auditory response in NS-affected dogs^48^, and other animal models have supported a link between noise-induced or genetic hearing loss and loudness intolerance^25,49^. It has also been proposed that dogs with NS might feel pain as a result of loud noise^50^ and many symptoms of NS do resemble the way dogs react to pain^16^.

Canine and human noise sensitivities appear to share similar features, but both genetic and morphological backgrounds of these conditions remain poorly characterized. Human sound sensitivity disorders include hyperacusis, misophonia and phonophobia^24,25,49^. Hyperacusis refers to decreased sound tolerance, in which the subjects can feel for example annoyance, fear, panic, rage, physical discomfort or pain due to sounds that are perceived as (too) loud. Misophonia is characterized by abnormally strong psychological reactions of annoyance or even rage towards context-related, soft or loud sounds, while in phonophobia certain sounds cause strong fear reactions. Sound intolerance is also a feature in some human neuropsychiatric conditions, such as autism spectrum disorders, in which auditory hypersensitivity is one of the major complications^51^.

The NS locus on chromosome 20 includes candidate genes that have been linked to human neuropsychiatric conditions and the hearing system. The candidate genes closest to the best-associated markers include the oxytocin receptor gene *OXTR*, a Rho GTPase gene *SRGAP3* and a metabotropic glutamate receptor gene *GRM7*.

*OXTR* encodes a receptor for oxytocin, which has been studied widely as a neural modulator of mammalian behaviour, including maternal, social and stress behaviours in both humans and dogs^52,53^. Studies also suggest that oxytocin and *OXTR* play a role in psychiatric disorders, such as anxiety disorders and autism^54–57^. In dogs, *OXTR* polymorphisms have been widely linked to human-directed social behaviour, greeting behaviour, proximity seeking and friendliness^58–60^ but so far not to NS or anxiety. *SRGAP3* regulates the actin cytoskeleton, is involved in neuronal development and, based on mouse models, is crucial for the normal cognitive behaviour^61^. *SRGAP3*, also known as ‘Mental disorder-associated GAP protein’ MEGAP, has been linked to severe mental retardation^62^ and a schizophrenia-related phenotype^63^.

*GRM7* encodes a G-protein-coupled metabotropic glutamate receptor subtype 7, known as mGlu7 or mGluR7^64^. The glutamate system, including mGlu7, seems to have a prominent role in anxiety-related behaviour and the link is supported by several genetic and functional studies^64–68^. In addition to the neuropsychiatric phenotypes, *GRM7* has been linked to age-related hearing loss and impairment in several studies^69^. Further downstream of the best-associated SNPs resides another hearing-related candidate gene, a plasma membrane calcium ATPase gene *ATP2B2* that plays a role in maintaining the Ca^2+^ balance vital for the proper functioning of the hearing sense^70^. PMCA2 dysfunctions have been linked to deafness and partial, noise-induced or progressive hearing loss in human and mice studies^71^, but also autism^70,72^, and it is suggested to have a role in nociception^73^. Since a role of possible hearing defects in canine NS has been suggested, the hearing-related genes lying in the NS associated locus could propose a novel target for future investigations.

The novel canine locus for fear against strangers and new situations on chromosome 7 is largely syntenic to human locus 18p11.2. This region has been repeatedly linked to psychiatric illnesses such as bipolar disease and schizophrenia^74,75^. Different anxiety disorders and fear are highly co-morbid with schizophrenia, but also with other psychiatric diseases, such as bipolar disorder^76^. The prevalence of anxiety disorders such as panic disorder, posttraumatic stress disorder, obsessive-compulsive disorder, generalized anxiety disorder and social anxiety disorder are increased among both bipolar and schizophrenia patients when compared to the general population^77,78^. Mapping canine fear to a locus syntenic to the human 18p11 that has been strongly linked to schizophrenia and bipolar disorder is an intriguing finding. It has been previously suggested that dogs with extremely fearful, shy and nervous behaviour towards unfamiliar situations or humans could share similarities with some symptoms or characteristics of schizophrenia^18^. Although the fearful dogs in this study exhibit normal variation of canine fearfulness rather than a pathological extremity, the finding suggests similar genetic background for canine fearfulness and some human psychiatric diagnoses with a strong component of anxiety and fear.

The CFA7 locus includes several interesting candidate genes. The genes include *GNAL*, a gene that encodes a stimulatory G-protein alpha subunit and has been linked to the dopaminergic neurotransmission pathway^79^. Interesting candidate genes also include *IMPA2*, a myoinositol monophosphatase gene that has been studied as a candidate for bipolar disorder^80^ and schizophrenia^81^. Three more genes in the region, metallophosphoesterase 1 (*MPPE1*), a SNAP-gamma gene *NAPG* and the vesicle-associated membrane protein-associated protein A (*VAPA*) have all been suggested to be candidate genes for bipolar disorder^82–84^. *NDUFV2*, encoding a subunit of the inner mitochondrial enzyme complex I, has been linked to bipolar disorder, major depression, schizophrenia and Parkinson’s disease^85,86^. Other possible, neuronally relevant candidate genes in the fear-associated locus in CFA7 that lie outside the human 18p11 locus include a myocin gene *MYO5B* that has been linked to schizophrenia and bipolar disorder^87,88^.

A single-SNP association was seen on chromosome 3 (at 13,525,675 bp) in the GWAS of fear. Closest genes to this isolated SNP include a pro-protein convertase gene *PCSK1* and an elongation factor gene *ELL2*, neither of which have been linked to anxiety in any species. This finding may represent a spurious association and requires a replication prior to further focus.

The genetics of canine behaviour have been studied using several differing approaches within or across breeds using clinic-, survey- or breed standard-based behavioural measures. Beside this study, successful examples with associations at genome-wide significance include canine compulsion^9,10^ and interspecies interaction^11^, while many other associations have remained at suggestive level. No significant associations have been reported to NS before our study, but a suggestive single variant association was reported by Ilska et al.^14^ Zapata et al.^13^ identified loci for fear and aggression in a cross-breed setup with averaged phenotypes, but the loci do not overlap with ours. The approach in Zapata et al. markedly differs from our within-breed analysis with individual phenotypic scores and the studies thus likely render two different views to the genetics of canine fear.

In summary, we have discovered two novel loci for canine anxiety. The loci harbour several relevant candidate genes that may contribute to predisposition to fear. Of particular interest are the genes that regulate glutamatergic and dopaminergic pathways and genes that affect the hearing system. Ongoing research aims to replicate the loci in larger cohorts and characterize the associated regions to find causative variants that would further facilitate the investigations into the molecular mechanisms. There is a current need for large animal models for the development of better anxiolytic drugs and fearful dogs may provide such natural models for human anxieties.

## Supplementary information

Legends for the supplementary tables and videos

Supplementary Table 1

Supplementary Table 2

Supplementary Table 3

Supplementary Table 4

Supplementary Video 1
