# Supplementary material for: Two novel genomic regions associated with fearfulness in dogs overlap human neuropsychiatric loci
Source: Transl Psychiatry. 2019 Jan 17;9:18. doi: 10.1038/s41398-018-0361-x (PMC6336819; doi:10.1038/s41398-018-0361-x)
Supplement: Supplementary file 1 — Legends for the supplementary tables and videos [file 41398_2018_361_MOESM1_ESM.docx]

**Supplementary information**

**Legends for the supplementary figures, tables and videos**

**Supplementary Table 1.** Noise reactivity score. A list of behavioural survey questions addressing dogs’ reactions towards noise were scored and used in calculating the noise reactivity score (NRS).

**Supplementary Table 2.** Fear reaction score. A list of behavioural survey questions addressing dogs’ reactions towards strange humans and novel situations were scored and used in calculating the fear reaction score (FRS).

**Supplementary Table 3.** Top 10 GWAS hits in noise sensitivity. The genome-wide SNP data of the noise sensitivity cohort was analysed by PLINK and GenABEL.

**Supplementary Table 4.** Top 10 GWAS hits in fear. The genome-wide SNP data of the fear cohort was analysed by PLINK and GenABEL.

**Supplementary Video 1.** Examples of behavioural responses and fear reactions of a non-fearful and fearful dog. The video demonstrates the reactions of a 2-year old non-fearful male German Shepherd and a 5-year old fearful female German Shepherd to a strange human and a novel object in a behavioural test situation. The dogs were selected for the behavioural test based on the results of the validated behavioural survey. The behavioural test situation seen in the video was not used as a phenotyping method in this study.
