# Supplementary figures and images for: Two novel genomic regions associated with fearfulness in dogs overlap human neuropsychiatric loci
Source: Transl Psychiatry. 2019 Jan 17;9:18. doi: 10.1038/s41398-018-0361-x (PMC6336819; doi:10.1038/s41398-018-0361-x)

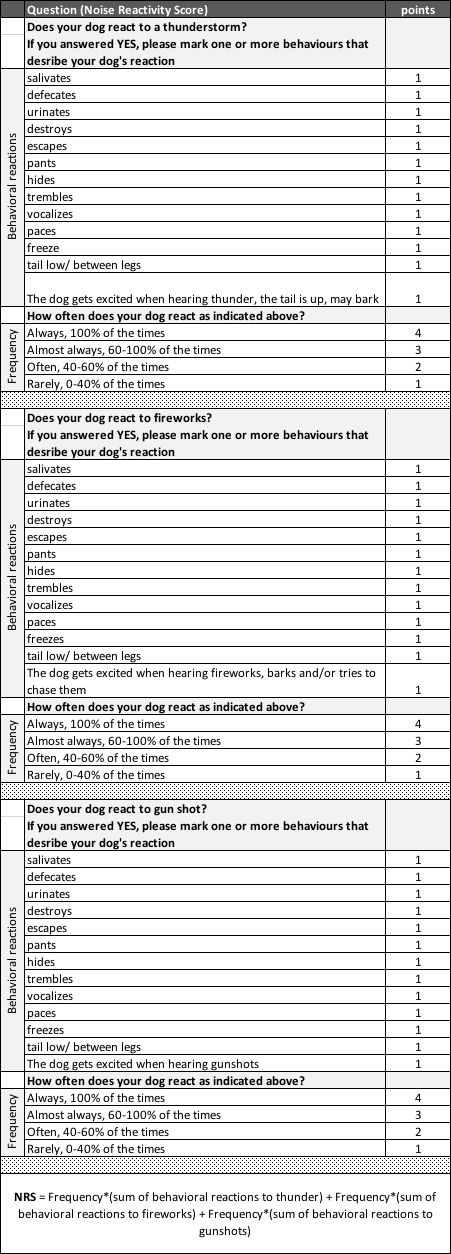

Supplement: Supplementary file 2 — Supplementary Table 1 [file 41398_2018_361_MOESM2_ESM.docx]

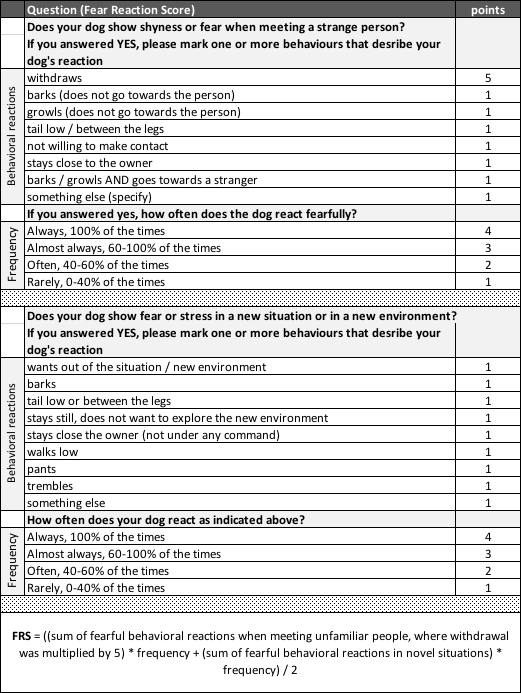

Supplement: Supplementary file 3 — Supplementary Table 2 [file 41398_2018_361_MOESM3_ESM.docx]
