# Supplementary material for: Two novel genomic regions associated with fearfulness in dogs overlap human neuropsychiatric loci
Source: Transl Psychiatry. 2019 Jan 17;9:18. doi: 10.1038/s41398-018-0361-x (PMC6336819; doi:10.1038/s41398-018-0361-x)
Supplement: Supplementary file 4 — Supplementary Table 3 [file 41398_2018_361_MOESM4_ESM.docx]

| PLINK: |  |  |  |
| --- | --- | --- | --- |
| **SNP NAME** | **CHR** | **POSITION** | **P-VALUE** |
| BICF2P252662 | 20 | 9'451'007 | 1,49E-07 |
| BICF2P1056282 | 20 | 9'731'317 | 1,06E-06 |
| BICF2G630229118 | 20 | 6'666'590 | 5,40E-05 |
| BICF2G630229151 | 20 | 6'680'138 | 5,40E-05 |
| BICF2G630229178 | 20 | 6'691'398 | 5,40E-05 |
| BICF2P860397 | 33 | 9'408'456 | 9,34E-05 |
| BICF2G630229120 | 20 | 6'668'293 | 0,0001359 |
| BICF2P1064605 | 20 | 10'192'388 | 0,0001421 |
| BICF2P1158072 | 20 | 10'478'868 | 0,0001472 |
| BICF2P1177517 | 20 | 10'517'467 | 0,0001472 |
|  |  |  |  |
| GenABEL: |  |  |  |
| **SNP NAME** | **CHR** | **POSITION** | **P-VALUE** |
| BICF2P252662 | 20 | 9'451'007 | 1.55E-07 |
| BICF2P1056282 | 20 | 9'731'317 | 5.46E-07 |
| BICF2G630229118 | 20 | 6'666'590 | 5.78E-05 |
| BICF2G630229151 | 20 | 6'680'138 | 5.78E-05 |
| BICF2G630229178 | 20 | 6'691'398 | 5.78E-05 |
| BICF2P1064605 | 20 | 10'192'388 | 7.032E-05 |
| BICF2P1158072 | 20 | 10'478'868 | 7.56E-05 |
| BICF2P1177517 | 20 | 10'517'467 | 7.56E-05 |
| BICF2P1264370 | 20 | 10'532'537 | 7.56E-05 |
| BICF2P617608 | 20 | 7'873'674 | 0,0001051 |
