# Supplementary material for: Two novel genomic regions associated with fearfulness in dogs overlap human neuropsychiatric loci
Source: Transl Psychiatry. 2019 Jan 17;9:18. doi: 10.1038/s41398-018-0361-x (PMC6336819; doi:10.1038/s41398-018-0361-x)
Supplement: Supplementary file 5 — Supplementary Table 4 [file 41398_2018_361_MOESM5_ESM.docx]

| PLINK: |  |  |  |
| --- | --- | --- | --- |
| **SNP NAME** | **CHR** | **POSITION** | **P-VALUE** |
| BICF2P862228 | 7 | 77'099'571 | 3,14E-09 |
| BICF2P205873 | 7 | 77'408'554 | 6,73E-09 |
| BICF2G630701448 | 3 | 13'525'675 | 1,14E-08 |
| BICF2P798404 | 7 | 76'487'265 | 1,08E-06 |
| BICF2P59496 | 7 | 77'639'464 | 2,72E-06 |
| BICF2S23637725 | 7 | 76'284'921 | 4,87E-06 |
| BICF2P428022 | 7 | 76'528'356 | 6,89E-06 |
| BICF2P1354669 | 7 | 76'829'221 | 7,64E-06 |
| BICF2P325158 | 7 | 75'935'406 | 1,13E-05 |
| BICF2G630754451 | 36 | 29'563'389 | 1,52E-05 |
|  |  |  |  |
| GenABEL: |  |  |  |
| **SNP NAME** | **CHR** | **POSITION** | **P-VALUE** |
| BICF2P862228 | 7 | 77'099'571 | 1.52E-08 |
| BICF2G630701448 | 3 | 13'525'675 | 1.93E-08 |
| BICF2P205873 | 7 | 77'408'554 | 2.56E-08 |
| BICF2P798404 | 7 | 76'487'265 | 2.39E-06 |
| BICF2P59496 | 7 | 77'639'464 | 5.90E-06 |
| BICF2S23637725 | 7 | 76'284'921 | 8.62E-06 |
| BICF2P428022 | 7 | 76'528'356 | 1.42E-05 |
| BICF2P1354669 | 7 | 76'829'221 | 1.44E-05 |
| BICF2G630754451 | 36 | 29'563'389 | 1.76E-05 |
| BICF2P325158 | 7 | 75'935'406 | 1.84E-05 |
